# Changes in the Quality of Life and Body Mass Index of Overweight Women after Interdisciplinary Interventions in Weight Reduction

**Published:** 2019-11

**Authors:** Malgorzata OBARA-GOLEBIOWSKA, Katarzyna Eufemia PRZYBYLOWICZ, Anna DANIELEWICZ, Jakub MORZE

**Affiliations:** 1. Department of Psychology of Development and Education, University of Warmia and Mazury, Olsztyn, Poland; 2. Department of Human Nutrition, University of Warmia and Mazury, Olsztyn, Poland

## Dear Editor-in-Chief

“Psychological practice in the scope of therapy of overweight persons shows that psychological destabilisation leading to a decrease in the quality of life of an individual frequently results in excessive eating” (1). Such a form of compensation directly results in overweight or obesity, and sometimes also in the appearance of deeper psychopathological disorders (2). On the other hand, weight reduction programs involving patient collaboration with physician, psychologist and dietitian lead to a significant increase in their quality of life (3).

The objective of this study was to analyse the changes in Body Mass Index (BMI) quality of life in a group of obese women of the Obesity Treatment Ward within 3 months after participating in multicomponent, interdisciplinary weight loss program.

The study group comprised 98 overweight females of the Obesity Treatment Ward which organizes weight loss programs that teach patients to make healthy lifestyle choices with the assistance of an interdisciplinary team of experts. All patients received a 1200 calorie diet. The applied research tool was The WHO Quality-of-Life Scale - WHOQOL-BREF. The study measured 3 times the BMI and quality of life of patients (question 1: individual overall quality of life; question 2: individual overall quality of health). The first measurement was made on the first day of the patient's stay in hospital. Measure second at the end of a two week weight-reduction program and the third measurement after 3 months. Measurements 1 and 2 were performed directly while measurement 3 was made via telephone or internet.

The mean BMI in the first measurement was 34.9 (SD +/− 6.0) with a mean BMI of 32.9 (SD +/− 5.9), a mean BMI of 30.9 (SD +/− 5.2) the third measurement. Significant differences applied to each pair of comparisons (*P*<0.05). The overall quality of life in the first measurement was 3.2 (SD +/− 0.8) versus 4.2 (SD +/− 0.9) in Measure II, 4.5 (SD +/− 0.6) in Measure III (Table 1). Significant differences are for each time point (*P*<0.05). The overall quality of health in the first measurement was 3.6 (SD +/− 0.7) against 4.0 (SD +/− 0.9) in Measure II, 4.3 (SD +/− 0.6) in Measure III ( Table 2).

**Table 1:** The WHOQOL-BREFF (overall quality of life)- ANOVA Friedman Test

***ANOVA Friedman***	***Average***	***Sum***	***Average***	***Standard deviation ***
Chi square ANOVA	Range	Range		
Overall quality of life I	1.7	73.0	3.3	0,8
Overall quality of life II	2.8	122.7	4.5	0,9
Overall quality of life III	3.2	138.7	4.7	0,6
Value p – post-hoc				
Overall quality of life I		0.000	0.000	0.000
Overall quality of life II	0.000		0.010	0.287
Overall quality of life III	0.000	0.010		0.000

A statistically significant (*P*=0.000) difference between overall quality of life (WHOQOL BREF) between I-III was observed.

**Table 2:** The WHOQOL-BREFF (overall quality of health)- ANOVA Friedman Test

***ANOVA Friedman***	***Average***	***Sum***	***Average***	***Standard deviation***
Chi square ANOVA (N = 98 , df 3 ) =41.7 *P*=0.001	Range	Range		
Overall quality of health I	2.2	90.2	3.8	0.7
Overall quality of health II	2.7	114.2	4.1	0.9
Overall quality of health III	3.1	132.2	4.3	0.6
Value p – post-hoc				
Overall quality of health I		0.000	0.000	0.000
Overall quality of health II	0.000		0.002	1.000
Overall quality of health III	0.000	0.002		0.002

A statistically significant (p = 0.000) difference was found between the overall quality of health (WHOQOL BREF) between measurements I-III. Significant differences were for each time point (*P*<0.05).

A 3-fold repetition test showed a statistically significant decrease in kilograms over a 3-month period. The results obtained are in line with the results of another study showing efficacy of multicomponent interventions (4). The results indicate that there is a need for multicomponent, interdisciplinary programs that target overweight people that teach them skills specific to the maintenance of weight loss. Multidisciplinary programs that involve frequent follow-up with providers and use a cognitive behavioral approach are associated with the greatest weight loss (5).

It is outlined that major components of such cognitive behavioral approaches, should focus on including self-monitoring, problem solving, nutrition education, stimulus control, cognitive restructuring, slowing down the rate of eating, and increasing exercise (6). Self-monitoring of physical activity and dietary intake (i.e., with a log), as well as monitoring of thoughts and emotions around food and eating can increase patients’ awareness of their problematic behaviors and cognitions and track progress.
